# In Vivo Chemical Reprogramming Is Associated With a Toxic Accumulation of Lipid Droplets Hindering Rejuvenation

**DOI:** 10.1111/acel.70390

**Published:** 2026-01-27

**Authors:** Wayne Mitchell, Cecília G. de Magalhães, Alexander Tyshkovskiy, Yushi Uchida, Ludger J. E. Goeminne, Takaharu Ichimura, Emery L. Ng, Alibek Moldakozhayev, Joseph V. Bonventre, Vadim N. Gladyshev

**Affiliations:** ^1^ Division of Genetics, Department of Medicine Brigham and Women's Hospital, Harvard Medical School Boston Massachusetts USA; ^2^ Division of Renal Medicine Brigham and Women's Hospital Boston Massachusetts USA; ^3^ Fralin Life Sciences Institute Virginia Tech Blacksburg Virginia USA

**Keywords:** aging, aging biomarkers, chemical reprogramming, lipid droplets, mitochondria, mitochondrial morphology, oxidative phosphorylation, rejuvenation, reprogramming

## Abstract

Partial reprogramming has emerged as a promising strategy to reset the epigenetic landscape of aged cells towards more youthful profiles. Recent advancements have included the development of chemical reprogramming cocktails that can lower the epigenetic and transcriptomic age of cells and upregulate mitochondrial biogenesis and oxidative phosphorylation. However, the ability of these cocktails to affect biological age in a mammalian aging model has yet to be tested. Here, we have characterized the effects of partial chemical reprogramming on mitochondrial structure and function in aged mouse fibroblasts and tested its in vivo efficacy in genetically diverse male UM‐HET3 mice. This approach increases the size of mitochondria, alters cristae morphology, causes an increased fusing of mitochondrial networks, and speeds up movement velocity. At lower doses, the chemical reprogramming cocktail can be safely administered to middle‐aged mice using implantable osmotic pumps, albeit with no effect on the transcriptomic age of kidney or liver tissues and only a modest effect on the expression of OXPHOS complexes. However, at higher doses, the cocktail causes a drastic reduction in body weight necessitating euthanasia. In the livers and kidneys of these animals, we observe significant increases in lipid droplet accumulation, as well as changes in mitochondrial morphology in the livers that are associated with mitochondrial stress. Thus, partial chemical reprogramming may induce mitochondrial stress and lead to significant lipid accumulation, which may cause toxicity and hinder the rejuvenation of cells and tissues in aged mammals.

## Graphical abstract

Partial chemical reprogramming significantly increases mitochondrial interaction networks, affects mitochondrial morphology, speeds up mitochondrial movement dynamics, increases mitochondrial transmembrane potential, and induces TCA cycle stress. These changes in mitochondrial function contribute to lipid droplet accumulation, acute kidney injury, and toxicity in vivo.

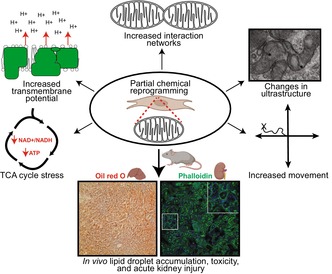

## 1 Introduction

Aging is characterized by underlying molecular changes that manifest as phenotypic declines in function and increased risk of mortality (López‐Otín et al. 2013). Aging displays considerable heterogeneity between different cell types, tissues, species, sexes, and even individual organisms of the same species and sex (Bou Sleiman et al. 2022; Ferrucci and Kuchel 2021; Hägg and Jylhävä 2021; Shadel et al. 2021; Yang, Harrison, and Promislow 2023). However, declines in the functioning of essential cellular processes such as proteostasis, mitochondrial bioenergetic function, and DNA repair and structural maintenance are often shared across these groups, colloquially forming the hallmarks of aging. While several molecular theories of aging have been proposed, such as epigenetic information loss (Lu, Tian, and Sinclair 2023), deleteriome (Gladyshev 2016; Gladyshev et al. 2021), antagonistic pleiotropy (Austad and Hoffman 2018), or hyperfunction (Blagosklonny 2012; Gems 2022), causal data that ties the therapeutic targeting of any of these biological features in question to a robust extension of mammalian lifespan is lacking. Given that directly intervening in the aging process could delay the onset of multiple aging‐related diseases in humans simultaneously (Sierra 2016), there is a pressing need to develop therapies that target aging despite our mechanistic insight shortcomings. One avenue that circumvents this issue is to focus on pharmacological, genetic, and dietary interventions that reduce biological age, which is more representative of health status and future risk of disease than chronological age (Li et al. 2023; Rutledge et al. 2022; Zurbuchen et al. 2025). More advanced transcriptomic biomarkers (Tyshkovskiy et al. 2019, 2024), epigenetic clocks (Belsky et al. 2022; Lu, Fei, et al. 2023; Lu et al. 2019; Ying et al. 2024), and proteomics predictors of mortality (Goeminne et al. 2025; Oh et al. 2023) are continually being developed that can accurately and unbiasedly assess the impact of an intervention on mammalian aging and lifespan (Lu et al. 2019; McCrory et al. 2020).

The discovery of the Yamanaka factors (Takahashi and Yamanaka 2006) has led to considerable interest in adapting the forced expression of *Oct4*, *Sox2*, *Klf4*, and *Myc* (OSKM) to rejuvenate aged cells while simultaneously preserving their cellular identity (Paine et al. 2024; Singh and Zhakupova 2022). Cyclic partial reprogramming with OSKM can extend the lifespan of progeria mice (Ocampo et al. 2016) and ameliorate some molecular aspects of aging in wild‐type C57BL/6J mice (Browder et al. 2022), but it can also lead to teratoma formation and toxicity in the liver and intestines as these tissues reprogram faster than others (Parras et al. 2023; Sahu et al. 2024). However, partial reprogramming with OSK alone has been shown to avoid these toxicity challenges, whilst still being able to restore vision in mouse models of glaucoma and aging and extend lifespan in wild‐type mice (Lu et al. 2020; Macip et al. 2024). Further efforts to translate OSK partial reprogramming to other model organisms and diseases are ongoing (Ksander et al. 2023; Pereira et al. 2024). One challenge that persists in developing partial reprogramming as an effective gene therapy is the adeno‐associated virus (AAV) delivery of exogenous OSK to various organs. For example, while AAV9 can effectively induce OSK expression in the heart and liver, it is unable to do so in the brain or pancreas (Macip et al. 2024). Thus, there is an unmet need to either develop AAVs capable of delivering genes to the vast majority of organs, or to develop alternative strategies that can partially reprogram cells and tissues that do not rely on AAV delivery.

Recent advancements have also led to the full reprogramming of human somatic cells to induced pluripotent stem cells (iPSCs) using multi‐stage chemical cocktail treatments (Guan et al. 2022; Liuyang et al. 2023; Wang et al. 2025). iPSCs generated through chemical reprogramming have further been differentiated to islet‐like cells and autologously transplanted into a patient with Type 1 diabetes, resulting in sustained improvements in glycemic control (Wang et al. 2024). Short‐term treatment, or partial chemical reprogramming, of fibroblasts with a subset of these compounds, in contrast, does not lead to de‐differentiation but instead can affect several molecular features associated with aging (Schoenfeldt et al. 2022). Furthermore, treatment with several of these cocktails can lower the transcriptomic age of both non‐senescent and senescent mouse and human fibroblasts, respectively (Mitchell et al. 2024; Yang, Petty, et al. 2023). Mechanistically, partial chemical reprogramming is associated with strong upregulation of mitochondrial biogenesis and oxidative phosphorylation (OXPHOS) and global DNA hypomethylation that also reduces cellular epigenetic age (Mitchell et al. 2024). However, it is still currently unknown if partial chemical reprogramming can affect biological age in other cell types and tissues, and the impact of partial chemical reprogramming on mitochondrial biology is not fully elucidated.

Therefore, to more fully characterize the mechanisms of partial chemical reprogramming, we analyzed the effects of 7c (repsox, tranylcypromine, valproate, forskolin, CHIR99021, DZNep, 4‐[(E)‐2‐(5,6,7,8‐Tetrahydro‐5,5,8,8‐tetramethyl‐2‐naphthalenyl)‐1‐propenyl]benzoic acid (TTNPB)25) treatment on mitochondrial morphology, interaction networks, and movement dynamics using microscopy. In tandem, we delivered the 7c cocktail to genetically diverse UM‐HET3 male mice to evaluate its ability to impact organismal biological age based on transcriptomic biomarkers in liver and kidney tissues. While lower doses were well‐tolerated, they did not lead to robust gene expression changes in the livers and kidneys. In contrast, higher doses led to rapid weight loss and lowered body scores requiring euthanasia. Staining of the tissues from the animals that received high doses of the 7c cocktail revealed a strong increase in lipid droplet formation, as well as increased abundance of liver mitochondria with abnormal morphologies. Taken together, our results suggest that the increase in lipid droplet formation during 7c partial chemical reprogramming may arise from mitochondrial metabolic stress and may lead to toxicity in vivo.

## 2 Materials and Methods

### 2.1 Materials and Animals

Cell culture media, consumables, and reagents were acquired from ThermoFisher Scientific (Waltham, MA, USA). All other general chemicals and reagents were purchased from Sigma‐Aldrich (St. Louis, MO, USA). Male C57BL/6J mice were acquired from the National Institute on Aging (NIA) aged rodent colonies (Charles River Laboratories, Wilmington, MA, USA). Male UM‐HET3 mice were purchased from Charles River Laboratories. Mice were given standard 5053 diet (LabDiet, St. Louis, MO, USA) and water ad libitum, were housed on a 12/12 h light/dark cycle at ~21°C, and were allowed to acclimate for at least 2 weeks prior to any experiments. C57BL/6J mice were housed in a specific‐pathogen free (barrier) facility at 5 animals per cage, whereas UM‐HET3 mice were housed in the in‐and‐out (non‐barrier) facility at 3 animals per cage. All experiments using mice were performed in accordance with institutional guidelines for the use of laboratory animals and were approved by the Brigham and Women's Hospital and Harvard Medical School Institutional Animal Care and Use Committees under Protocol no. 2016N000368. Mouse ear fibroblasts were isolated from 25‐month‐old male C57BL/6J mice as previously described (Mitchell et al. 2024) and were used exclusively at low passage numbers for all assays (< 5). Chemical reprogramming compounds were obtained from the vendors listed in Table S1 and were used at the concentrations listed. Analgesics and other surgery supplies for the osmotic pump implantations were acquired from Patterson Veterinary Supply (Loveland, CO).

### 2.2 Embedding, Sectioning, and TEM Imaging

25‐month‐old mouse ear fibroblasts were treated with 7c or 50:50 PBS: DMSO vehicle for 6 days and cultured at 37°C, 5% CO_2_, and 3% O_2_ in DMEM/F12 supplemented with 10% FBS, 1× antibiotic and antimycotic, 1× non‐essential amino acids, and 50 μM β‐mercaptoethanol. Media and treatments were replaced once after 3 days. Cells were then seeded onto UV‐sterilized Aclar plastic coverslips in 24‐well plates and grown overnight at a starting cell density of 100,000 cells per well. After aspirating the media and rinsing once with PBS, cells were fixed in 2.5% glutaraldehyde, 1.25% paraformaldehyde, and 0.3% picric acid in 0.1 M sodium cacodylate buffer (pH 7.4) for 1 h at room temperature. Then, the cells were washed in 0.1 M sodium cacodylate buffer (pH 7.4), post‐fixed for 30 min in 1% osmium tetroxide and 1.5% potassium ferrocyanide, and washed twice in water and once in maleate buffer. Following this, cells were incubated in 1% uranyl acetate in maleate buffer for 30 min, followed by two washes with water and subsequent dehydration in grades of alcohol (5 min each: 50%, 70%, 95%, and 2 × 100%). Finally, the samples were embedded in TAAB Epon (TAAB Laboratories Equipment Ltd., Berkshire, England) and polymerized at 60°C for 48 h.

After polymerization, the Aclar plastic was peeled off, and a small area (~1 mm) of the flat embedded cells was cut out with a razor blade and remounted on an Epon block. Ultrathin sections (~80 nm) were cut on a Reichert Ultracut‐S microtome, picked up onto copper grids stained with lead citrate, and examined on a TecnaiG^2^ Spirit BioTWIN. Images were taken using an AMT 2k CCD camera. Measurements of mitochondrial size were performed manually in FIJI (Schindelin et al. 2012).

### 2.3 Oil Red O Staining

25‐month‐old mouse ear fibroblasts were treated with 2c (repsox, tranylcypromine), 7c, or 50:50 PBS: DMSO vehicle for 6 days and cultured at 37°C, 5% CO_2_, and 3% O_2_ in DMEM/F12 supplemented with 10% FBS, 1× antibiotic and antimycotic, 1× non‐essential amino acids, and 50 μM β‐mercaptoethanol. Media and treatments were replaced once after 3 days. Cells were then seeded in 24‐well plates and grown overnight at a starting cell density of 100,000 cells per well. Oil red O stock solution was prepared by dissolving 60 mg in 20 mL of 100% isopropanol and incubating at room temperature for 30 min. A working solution of Oil red O was prepared by mixing 6 mL of stock solution with 4 mL of water, incubating at room temperature for 10 min, and filter sterilizing through a 0.22‐μm filter. After aspirating the media, the cells were washed twice with PBS prior to fixation in 4% paraformaldehyde for 30 min on a rotator at room temperature. Following this, the cells were rinsed once with PBS, incubated for 5 min in 60% isopropanol, and then stained with Oil red O for 15 min. After rinsing five times with PBS, the cells were imaged on an EVOS microscope (ThermoFisher Scientific, Waltham, MA, USA). Following image acquisition, the PBS was aspirated and replaced with 200 μL of 100% isopropanol. After mixing and incubating for 2 min at room temperature, the solutions were withdrawn and added to a clear 96‐well plate, and the absorbances were read at 492 nm on a BioTek Synergy H1 microplate reader (Agilent Technologies, Lexington, MA, USA).

### 2.4 Osmotic Pump Implantations

Osmotic minipump models 1004 and 2004 were purchased from DURECT Corporation (Cupertino, CA, USA). Male UM‐HET3 mice were anesthetized with isoflurane using a precision vaporizer (SomnoFlo Suite, Kent Scientific) and primed pumps filled with either vehicle (50% *v*/*v* DMSO/PEG300) or 7c (valproate, DZNep, forskolin, TTNPB, tranylcypromine, repsox, CHIR99021) were randomly assigned and subcutaneously implanted for 28 days. To account for any potential cage effects, mice were housed individually after the procedure. Following 28 days of treatment, mice were euthanized by CO_2_ and cervical dislocation, after which blood and tissues were collected and either fixed in 4% paraformaldehyde overnight or flash‐frozen in liquid nitrogen and stored at −80°C.

### 2.5 Confocal Microscopy

For live cell imaging, 50,000 cells were seeded overnight on 35 mm Ibidi dishes with #1.5 polymer coverslips and stained for 30 min with 50 nM TMRM, 100 nM MitoView Green, and 10 μg/mL Hoechst 33342 in live cell imaging media (fibroblast growth media with 4 mM L‐glutamine and without phenol red). Cells were then imaged on a CSU‐W1 Yokogawa spinning disk with a 50 μm pinhole disk and an Andor Zyla 4.2 Plus sCMOS monochrome camera built around an inverted Nikon Ti microscope equipped with a heated enclosure and CO_2_ control. Cells were imaged with either a 20×/0.75 air or 100×/1.45 oil objective. Fluorescence intensities were quantified in FIJI (Schindelin et al. 2012), and mitochondrial morphology networks were analyzed using the Mitochondria Analyzer (MitoAnalyzer) plug‐in (Chaudhry et al. 2020) on the TMRM images. Time‐lapse mitochondrial morphology and dynamics were quantified using Mitometer (Lefebvre et al. 2021).

For imaging of fixed cells, 20,000 cells per well were seeded overnight on Nunc Lab‐Tek II 8‐chambered #1.5 coverglass that were coated with 0.2% gelatin for at least 1 h prior to cell seeding. Cells were then rinsed with PBS, fixed in 4% paraformaldehyde, and permeabilized with 0.3% Triton X‐100, all at room temperature. After blocking with 5% bovine serum albumin (BSA) for 30 min, cells were stained with 1:200 mouse anti‐Tom20 primary antibody (Novus Biologicals, Centennial, CO, USA) for 1 h, followed by 1:200 rabbit anti‐mouse AlexaFluor 568 secondary antibody (ThermoFisher Scientific, Waltham, MA, USA) for 1 h and 1 μg/mL DAPI for 10 min, all at room temperature before three final washes in PBS. Cells were imaged using a Zeiss point scanning LSM980 confocal built around a Zeiss Axio Observer Z1 and equipped with two multi‐alkali PMTs and a GaASP 32 channel spectral detector, an environmental enclosure, and a 63×/1.4 oil objective. 3D (*z*‐stack) images with either 0.2‐ or 0.5‐μm step sizes of mitochondrial morphology networks were analyzed using the MitoAnalyzer FIJI plug‐in (Chaudhry et al. 2020).

### 2.6 Tissue Sectioning and Staining

Fresh tissues were rinsed several times in PBS before fixing in 4% paraformaldehyde overnight at 4°C with gentle rotation. Then, tissues were cut with a razor blade and either infiltrated with 30% sucrose overnight or embedded in paraffin and cut into 5‐μm sections on a microtome. The sucrose‐infiltrated tissues were then frozen in OCT blocks and cut into 20‐μm sections on a cryostat.

Paraffin‐embedded sections were stained either with hematoxylin and eosin or Masson's trichrome according to standard, published procedures. OCT‐embedded sections were stained with Oil red O and counterstained with hematoxylin. Stained tissues were imaged on an Axio Imager 2 (Zeiss, Oberkochen, Baden‐Württemberg, Germany) using an EC Plan‐Neofluar 10×/0.3 M27 objective and an Axiocam 105 color camera. The percent of tissue stained with Oil red O for control and partially chemically reprogrammed tissues was determined by using the Trainable Weka Segmentation FIJI plug‐in (Arganda‐Carreras et al. 2017). Cellular and nuclear structures of hematoxylin‐ and eosin‐stained images were determined using QuPath (Bankhead et al. 2017).

Frozen kidney sections were hydrated in PBS and treated with 1% SDS in PBS for 5 min. After blocking with 3% BSA in PBS, the sections were incubated with primary antibodies for 1 h at room temperature. Goat polyclonal anti–KIM‐1 (R&D Systems, Minneapolis, MN, USA) was used as the primary antibody. For co‐staining, biotinylated *Lotus tetragonolobus* lectin (LTL; Vector Laboratories, Newark, CA, USA) was applied to detect proximal tubule brush borders. The sections were then incubated with Cy3‐conjugated anti‐goat IgG as the secondary antibody and streptavidin‐DyLight 488 for 30 min at room temperature. For phalloidin staining, the sections were incubated with FITC‐conjugated phalloidin (ThermoFisher Scientific, Waltham, MA, USA) for 30 min at room temperature. After final washes, the slides were mounted using Vectashield mounting medium containing DAPI (Vector Laboratories, Newark, CA, USA), and images were acquired using a Leica Stellaris 5 confocal microscope (Leica Microsystems, Wetzlar, Germany). Phalloidin‐stained tissues were imaged less than a week after staining in a single imaging session.

Frozen liver sections were washed three times for 10 min in PBS, then blocked for 1 h at room temperature in 5% BSA with 0.1% Triton X‐100 in PBS. Following blocking, sections were incubated overnight at 4°C in a humidified chamber with the primary antibodies rabbit anti‐Tom20 (1:300, Novus Biologicals, NBP2‐67501) and mouse anti‐PMP70 (1:300, Sigma‐Aldrich, SAB4200181) diluted in PBS containing 0.25% Triton X‐100. After primary antibody incubation, sections were washed three times for 5 min each in PBS and subsequently incubated overnight at 4°C with Alexa Fluor 647‐conjugated anti‐rabbit (1:500, ThermoFisher Scientific, A21244) and Alexa Fluor 568‐conjugated anti‐mouse (1:500, ThermoFisher Scientific, A11001) secondary antibodies diluted in PBS. After secondary antibody incubation, sections were washed three times for 10 min each in PBS, then incubated for 30 min at room temperature with BODIPY 493/503 (1:1000, MedChem Express, HY‐W090090) and DAPI (1:1000, Sigma‐Aldrich, D9542) diluted in PBS. Finally, sections were washed again three times for 10 min each in PBS, mounted with ProLong Glass Antifade Mountant (Invitrogen, P36980), and stored at 4°C overnight before imaging.

For each liver sample, three randomly selected regions were imaged using a Nikon Ti2 inverted spinning disk confocal microscope equipped with an Andor Zyla 4.2 Plus sCMOS monochrome camera. Images were acquired using a Plan Apo λ 100×/1.45 oil objective. Laser power, exposure time, and threshold settings were kept constant across all samples to ensure comparability. For each region, a complete *z*‐stack was collected at 0.9‐μm intervals. Images were processed in FIJI (Schindelin et al. 2012) to generate two‐dimensional projections and to apply linear brightness and contrast adjustments. To quantify donut‐shaped mitochondria, individual cells were manually segmented. Linear contrast enhancement and background subtraction were applied to improve the signal‐to‐noise ratio of the Tom20 staining. Mitochondria were automatically detected in CellProfiler version 4.2.6 (Stirling et al. 2021) using the Otsu adaptive thresholding method, allowing quantification of the total number of mitochondria per cell. Donut‐shaped mitochondria were then manually counted, as automated segmentation and shape‐based classification algorithms were unable to discriminate this morphology from other mitochondrial forms.

### 2.7 RNA‐Seq

RNA from ~30 mg of flash‐frozen tissue was purified using a direct‐zol RNA miniprep kit (Zymo Research, Irvine, CA, USA) after tissue homogenization using a Bead Ruptor Elite homogenizer (OMNI International, Kennesaw, GA, USA) and 2‐mL tubes pre‐filled with 2.8‐mm ceramic beads. RNA concentration was measured by Qubit using the RNA HS assay kit, and library prep and sequencing were performed as described previously (Mitchell et al. 2025). Reads were mapped to the mouse genome (GRCm39) with STAR (version 2.7.11b) and counted via featureCounts (version 2.0.6). To filter out non‐expressed genes, we left only genes with at least 10 reads in at least 50% of samples separately for liver and kidney datasets. Differentially expressed genes were identified separately for each tissue using a one‐way ANOVA model through the edgeR package (Robinson et al. 2010).

### 2.8 Transcriptomic Signature Analysis

To explore how transcriptomic alterations induced by 7c treatment relate to known molecular signatures of aging, lifespan regulation, and reprogramming, we conducted functional enrichment analysis. We utilized reference datasets encompassing tissue‐specific aging signatures from liver, kidney, and brain, as well as multi‐tissue biomarkers of biological age and expected mortality, adjusted for chronological age (Mitchell et al. 2025; Tyshkovskiy et al. 2023). Additionally, we included a hepatic signature of expected maximum lifespan in rodents (Tyshkovskiy et al. 2024), along with transcriptional profiles of iPSC reprogramming via OSKM factor induction in mice and across species (Kriukov et al. 2022). For each tissue (liver and kidney), we ranked genes using a signed log‐transformed *p*‐value metric estimated through differential expression analysis:

$$-\log\left(\mathrm{pv}\right)\times \mathrm{sgn}\left(\mathrm{lfc}\right)$$

where *pv* and *lfc* are *p*‐value and logFC of a certain gene, respectively, and sgn is the signum function (equal to 1, −1, and 0, if value is positive, negative, or equal to 0, respectively). Ranked gene lists were then subjected to gene set enrichment analysis (GSEA) using the fgsea package in R, with 10,000 permutations and multilevel Monte Carlo sampling. Gene sets were drawn from the HALLMARK, KEGG, and REACTOME collections of the Molecular Signatures Database (MSigDB). The same enrichment pipeline was applied to reference signatures of aging, mortality, maximum lifespan, and iPSC reprogramming. To quantify similarities between 7c‐induced and reference gene expression biomarkers, we computed Spearman correlations between normalized enrichment scores (NES).

### 2.9 Transcriptomic Age Analysis

The filtered RNA‐seq data were processed with Relative Log Expression (RLE) normalization, log‐transformation, and scaling. Missing expression values for clock genes not detected in the dataset were imputed using their corresponding precomputed average values. Normalized gene expression profiles were centered to the median profile of control samples. Transcriptomic age (tAge) for each sample was estimated using Bayesian Ridge multi‐tissue transcriptomic clocks of chronological age and expected mortality (Tyshkovskiy et al. 2024). Module‐specific transcriptomic clocks of chronological age were applied to scaled relative gene expression profiles of control, 2c‐, and 7c‐treated cells identified previously (Mitchell et al. 2024) using the same framework.

### 2.10 Proteomics and Phosphoproteomics Analysis

To compare differential protein abundance and differential phosphorylation of 2c versus 7c, we re‐analyzed our TMT proteomics and phosphoproteomics datasets described previously (Mitchell et al. 2024), with the same msqrob2 (Goeminne et al. 2016, 2018) model structure that includes the treatment (control, 2c, or 7c), age group (4 months, or 20 months), and the treatment: age group interaction as covariates. We also reused the GSEA described previously (Mitchell et al. 2024). For the targeted phosphoprotein enrichment analysis in Figure S2C, we performed GSEA on the protein‐level enrichments from the phosphopeptides (Mitchell et al. 2024) with only the four gene sets: “Structural constituent of muscle (Gene Ontology identifier (GO): 0008307),” “Lipolysis in adipocytes (Kyoto Encyclopedia of Genes and Genomes identifier (KEGG): mmu04923),” “Mitochondrion (GO: 0005739),” and “mRNA splicing, via spliceosome (GO: 0000398).”

### 2.11 Western Blotting

Approximately 30‐mg pieces of flash‐frozen liver tissues were homogenized in 60 mM Tris–HCl, pH 6.8, 5% SDS buffer in 2‐mL tubes pre‐filled with 2.8‐mm ceramic beads on a Bead Ruptor Elite homogenizer (OMNI International, Kennesaw, GA, USA). Following homogenization, samples were spun at 12,000× *g* for 2 min and the protein concentration of the supernatants was measured by BCA assay using a BioTek Synergy H1 microplate reader (Agilent Technologies, Lexington, MA, USA). 15 micrograms of protein were loaded onto TGX 4%–15% gradient gels (Bio‐Rad, Hercules, CA, USA) and run at 120 V for ~1 h, transferred to PVDF membranes, and blotted for β‐actin (Santa Cruz Biotechnology, Dallas, TX, USA) or OXPHOS subunits (ThermoFisher Scientific, Waltham, MA, USA). Blots were visualized by staining with IRDye 680RD secondary antibody and imaging on a LI‐COR Odyssey Fc molecular imager (Lincoln, NE, USA).

### 2.12 Measurement of Cellular ATP, NAD ^+^, and NADH

Cellular ATP, NAD+, and NADH levels were measured using luminescence‐based detection kits from Promega (Madison, WI, USA). Luminescence was measured in white opaque 96‐well plates on a BioTek Synergy H1 plate reader (Agilent Technologies, Santa Clara, CA, USA).

### 2.13 Measurement of Cellular Lysosome Content

Live cells were seeded onto 35 mm Ibidi dishes as described previously and stained with 50 nM LysoTracker Green, 50 nM TMRM, and 10 μg/mL Hoechst 33342 in live cell imaging media for 20 min at 37°C, 5% CO_2_, and 3% O_2_ before imaging. Cells were imaged on an Axio Observer 7 using a Plan‐Apochromat 40×/1.3 oil objective. Lysosomal and mitochondrial areas were measured using FIJI (Schindelin et al. 2012) after rolling ball background subtraction and Otsu thresholding.

### 2.14 Statistical Analysis

For the proteomics and phosphoproteomics analysis, we used the msqrob2 and GSEA analyses described before (Mitchell et al. 2024). For comparison of transcriptomic ages (tAges), predicted by composite clocks, a mixed‐effects ANOVA model was implemented via the rma.uni function from the metafor package in R. For comparison of tAges estimated by module‐specific clocks, a one‐way ANOVA was used. For omics analyses, *p*‐values were adjusted for multiple testing with the Benjamini–Hochberg method (Benjamini and Hochberg 1995). In other cases, *p*‐values for comparisons between control and partially reprogrammed groups were determined by two‐tailed unpaired or paired *t*‐tests assuming equal variance. Statistical tests were performed using either the R package limma (Ritchie et al. 2015) or GraphPad Prism version 10.2.3. All measurements consist of at least *n* = 3 independent biological replicates with their corresponding means (bar height) and standard deviations (error bars) depicted in the figures.

## 3 Results

### 3.1 Partial Chemical Reprogramming With the 7c Cocktail Affects Mitochondrial Bioenergetics, Networks, Dynamics, and Morphology in Fibroblasts

**FIGURE 1:**
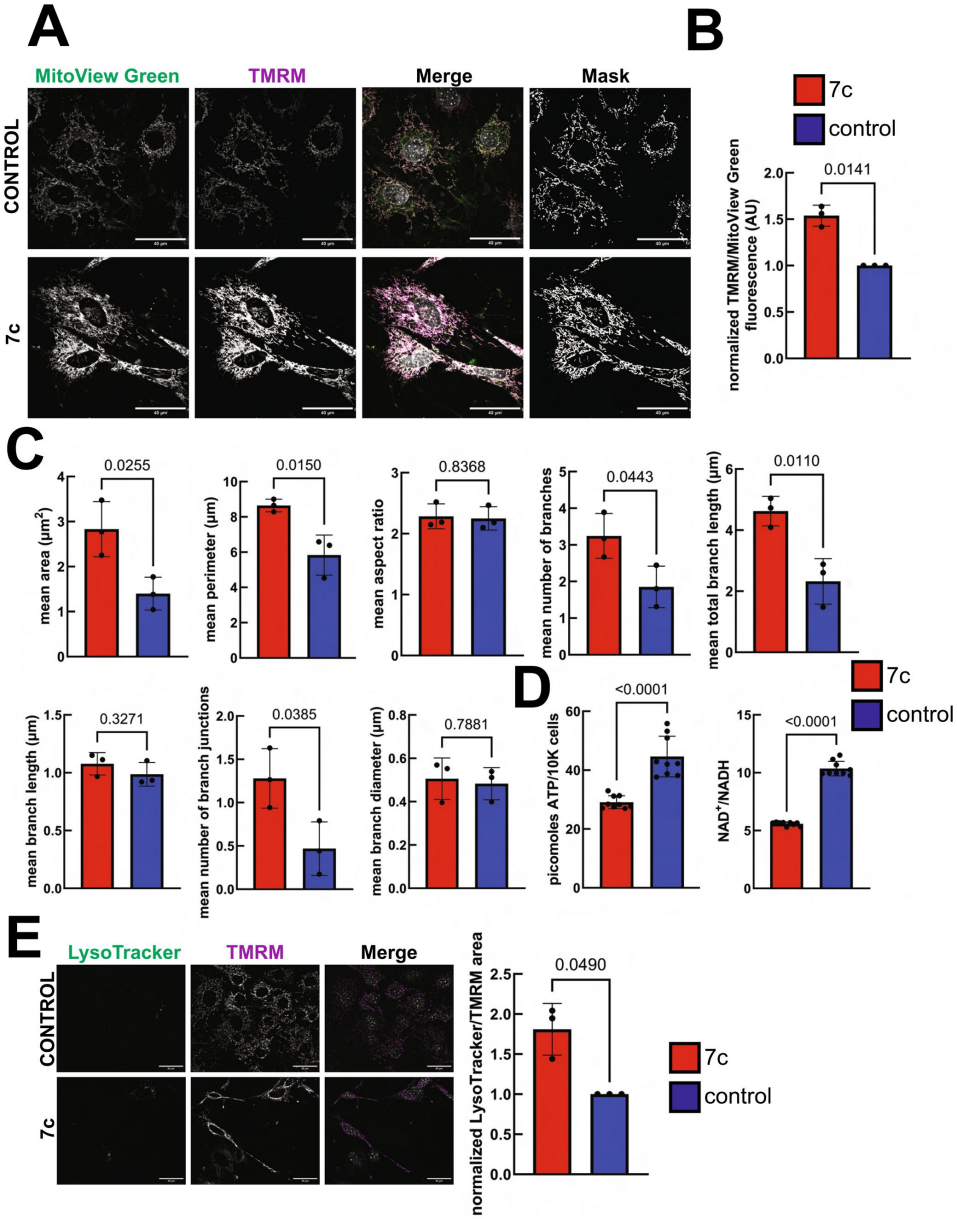
Effect of 7c treatment on mitochondrial 2D morphology and function in mouse fibroblasts. (A) MitoView Green and TMRM staining in live cells. 2D masks (right panels) were generated by thresholding using the MitoAnalyzer FIJI plug‐in (Chaudhry et al. 2020). Scale bars: 40 μm. (B) Quantification of mitochondrial transmembrane potential. TMRM signal was normalized to account for changes in mitochondrial mass by dividing by the MitoView Green signal. Error bars represent sample means ± standard deviation (SD). *p*‐value was determined by two‐tailed paired *t*‐test. *n* = 3 independent biological replicates. (C) Measured mitochondrial 2D parameters. *p*‐values were determined by two‐tailed unpaired *t*‐test. *n* = 3 independent biological replicates per treatment. Error bars represent sample means ± SD. (D) Cellular ATP, NAD^+^, and NADH levels. *p*‐values were determined by two‐tailed unpaired *t*‐test. *n* = 3 independent biological replicates per group, with 3 technical replicates for each biological replicate. Error bars represent sample means ± SD. (E) Cellular lysosome content. Live cells were stained with TMRM and LysoTracker Green (left panels). Scale bars: 40 μm. Lysosome content was quantified by normalizing LysoTracker area to TMRM area (right panel). Error bars represent sample means ± SD. *p*‐value was determined by two‐tailed paired *t*‐test. *n* = 3 independent biological replicates per treatment.

Previously, we reported that partial chemical reprogramming with only the 7c and not the 2c (repsox, tranylcypromine) cocktail drove a significant increase in OXPHOS expression and mitochondrial spare respiratory capacity (Mitchell et al. 2024). Therefore, we further investigated the effects of 7c treatment on mouse fibroblast mitochondrial function, morphology, and interaction networks using live‐cell imaging. Co‐staining fibroblasts with mitochondrial transmembrane potential‐independent (MitoView Green) and ‐dependent (TMRM) dyes revealed that 7c increased the fluorescence intensity of both compared to control cells treated with vehicle (Figure 1A, left panels). Moreover, we used the TMRM signal to construct 2D masks of the mitochondrial networks (Figure 1A, right panels). When we normalized the TMRM signal to account for changes in mitochondrial mass detected by MitoView Green, we still observed a significant increase in the mitochondrial transmembrane potential following 6 days of 7c treatment (Figure 1B). We then used the 2D masks to measure the effects of partial chemical reprogramming with 7c on mitochondrial size and interaction networks. Following 7c treatment, we observed significant increases in mitochondrial area and perimeter, in addition to the number of branches, total branch length, and branch junctions (Figure 1C). To see if these observations coincided with changes in TCA cycle function, we measured ATP, NAD^+^, and NADH levels in control‐ and 7c‐treated cells (Figure 1D). Contrary to expectation, 7c treatment lowered ATP levels and the cellular NAD^+^/NADH ratio, which is an indicator of cellular metabolic health and redox state (Lin and Guarente 2003; Williamson et al. 1967). Since 7c treatment may be inducing mitochondrial stress as a result of increased OXPHOS activity, we then stained live cells for lysosomes with LysoTracker Green (Figure 1E). We found that 7c treatment causes increased lysosome formation as determined by the normalized ratio of LysoTracker to TMRM area (Figure 1E, right), which may be in response to increased mitophagy of stressed and/or dysfunctional mitochondria.

**FIGURE 2:**
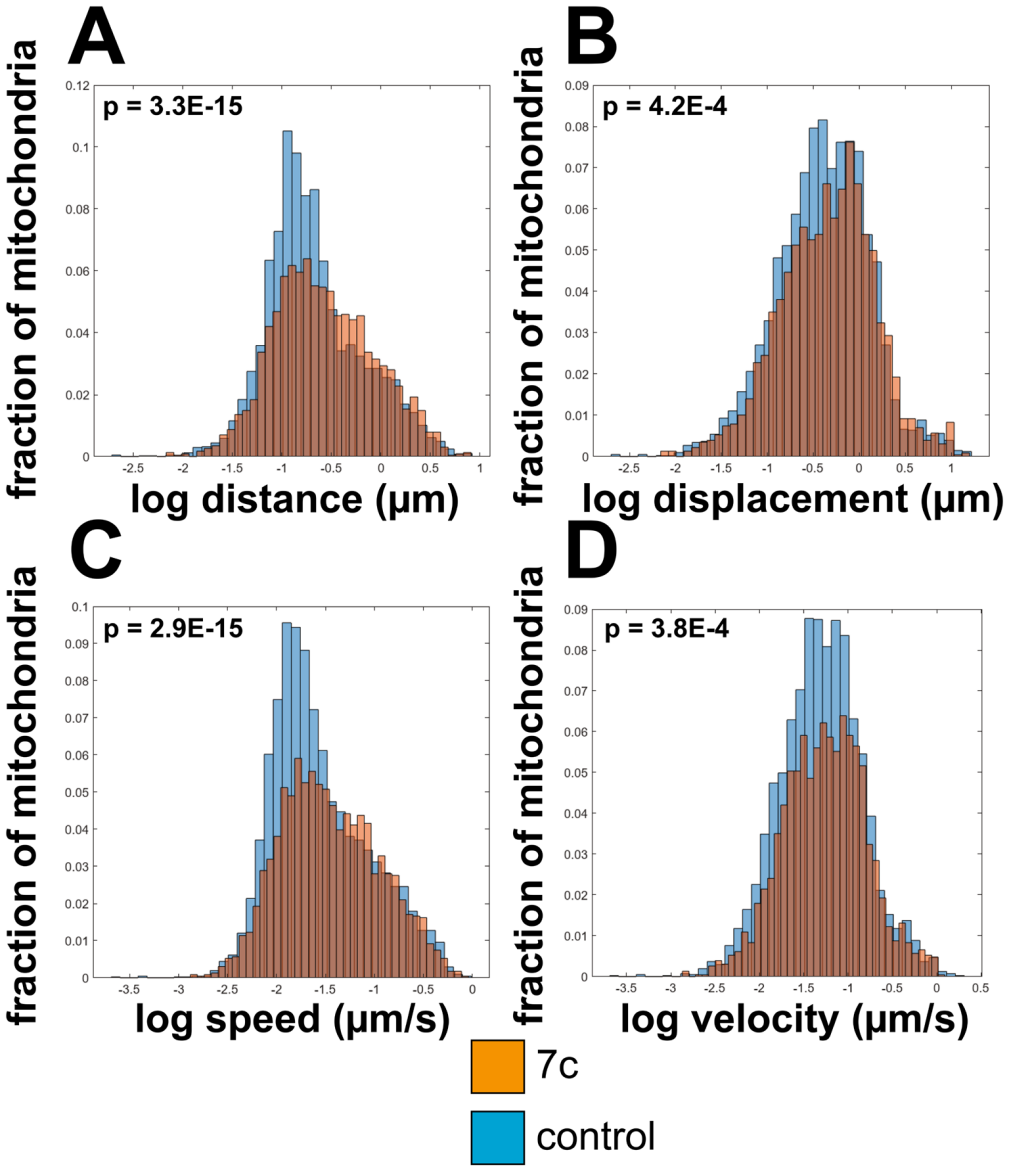
Effect of 7c treatment on mitochondrial 2D dynamics. (A) Distance. (B) Displacement (C) Speed. (D) Velocity. *p*‐values were determined by one‐way ANOVA using the Mitometer MATLAB plug‐in (Lefebvre et al. 2021). Data represents *n* = 15 movies per treatment group across 3 independent biological replicates (5 movies for each biological replicate).

We further collected time‐lapse TMRM images (10 s intervals, 3 min total) and analyzed them to determine the impact of partial chemical reprogramming on mitochondrial movement dynamics. Relative to control cells, we observed that 7c treatment increased the average total distance traveled (Figure 2A) and displacement (distance from starting position) (Figure 2B) of the mitochondria, which was further supported by significant increases in mitochondrial movement speed (rate of movement) (Figure 2C) and velocity (rate of movement in a given direction) (Figure 2D).

**FIGURE 3:**
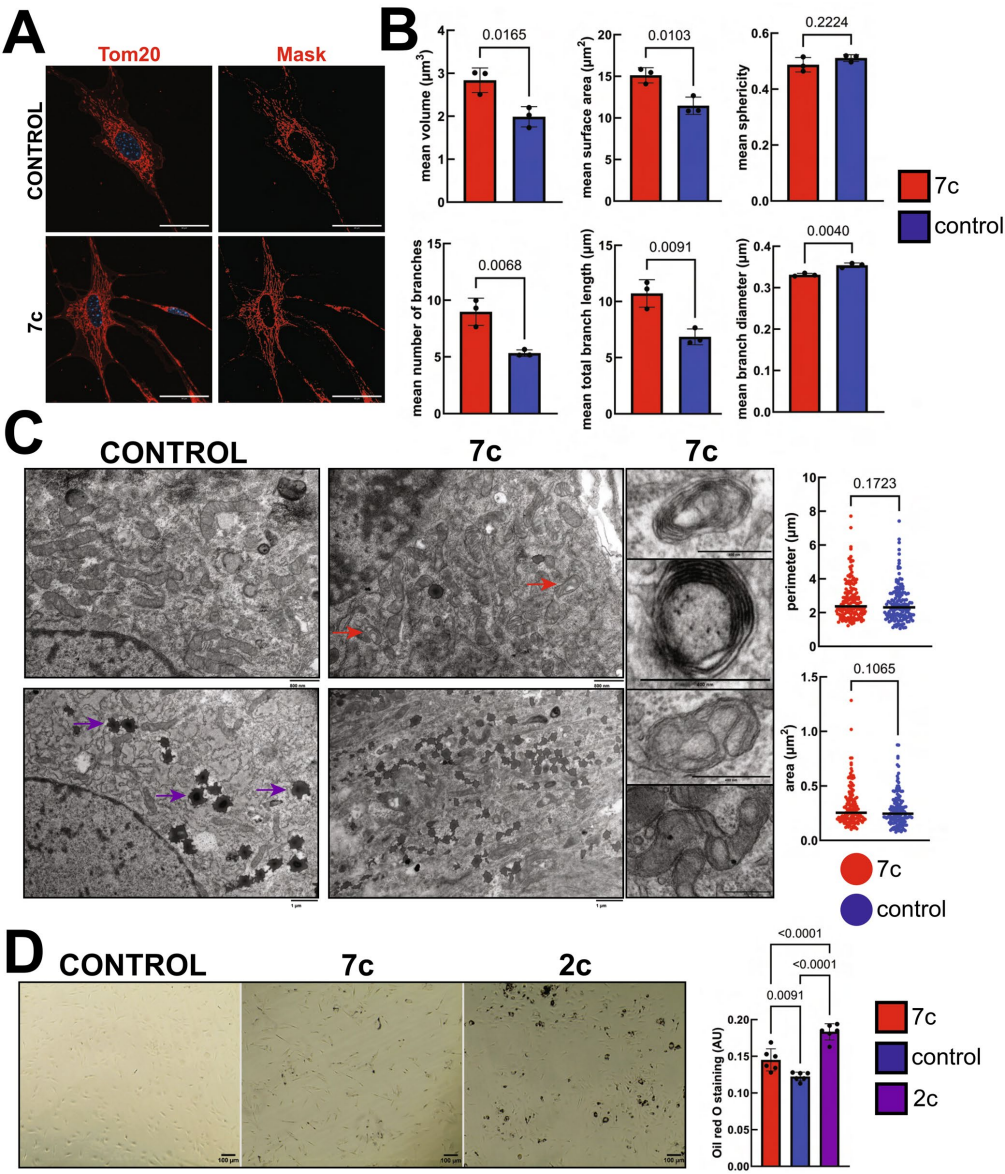
Effect of 7c treatment on mitochondrial 3D morphological networks, cristae morphology, and lipid droplet formation. (A) 3D projections of Tom20 staining. Maximum intensity projections of Tom20 staining (left panels) and 3D projections of masks (right panels) generated using the MitoAnalyzer FIJI plug‐in (Chaudhry et al. 2020). Scale bars: 40 μm. *Z*‐stacks were obtained using a 0.2‐μm step size. (B) Measured 3D mitochondrial parameters. *p*‐values were determined by two‐tailed unpaired *t*‐test. *n* = 3 independent biological replicates per treatment. Error bars represent sample means ± SD. (C) TEM images of lead citrate‐stained ultrathin fibroblast cell sections. Red arrows indicate mitochondria with abnormal/circular cristae morphology. Purple arrows indicate example lipid droplets. Single examples of atypical cristae morphology are shown to the right (scale bars: 400 nm). Bars in graphs to the right represent sample means. *p*‐values were determined by two‐tailed unpaired *t*‐test. *n* = 100 randomly selected mitochondria per treatment from images taken of 3 independent biological replicates. (D) Oil red O staining. Brightfield images of cells stained with Oil red O (left panels), and quantification of amount of Oil red O staining based on absorbance at 492 nm (right panel). Scale bars: 100 μm. *p*‐values were determined by one‐way ANOVA and Tukey's post hoc test. *n* = 6 independent biological replicates. Error bars represent sample means ± SD.

To more precisely measure the effect of 7c partial chemical reprogramming on mitochondrial morphology, we fixed and stained cells for Tom20 and collected 0.2 μm z‐stacks. We then generated 3D masks of the mitochondrial networks using the MitoAnalyzer FIJI plug‐in (Chaudhry et al. 2020) (Figure 3A). Control experiments using pre‐treatment with a mitochondrial uncoupler (CCCP, Figure S1) demonstrated that dissipation of the mitochondrial transmembrane potential significantly decreases mitochondria size, induces the formation of more spherical mitochondria, and disrupts mitochondrial branching networks. Following 7c treatment, we observed significant increases in mitochondrial volume, surface area, number of branches, and total branch length (Figure 3B). Taken together, these data further verify that partial chemical reprogramming induces significant effects on mitochondrial function, morphology, and dynamics.

### 3.2 7c Treatment Affects Cristae Morphology and Increases Lipid Droplet Accumulation in Fibroblasts

As cristae structure and density is closely associated with mitochondrial bioenergetic function (Baker et al. 2019), we then used transmission electron microscopy (TEM) to ascertain the impact of 7c treatment on cristae morphology (Figure 3C). In control cells, we observed typical cristae morphology characterized by tightly stacked, thin lamellae that fully cross the mitochondrial minor axes. However, with 7c‐treated cells, we noticed a larger proportion of mitochondria with circular and/or onion‐like cristae (red arrows). Although not statistically significant, we also saw a trend towards increased mitochondrial area and perimeter following 7c treatment. Unexpectedly, we also observed an increase in the number of lipid droplets (purple arrows) following partial chemical reprogramming. We validated our findings by quantifying Oil red O staining in control‐, 2c‐, and 7c‐treated cells (Figure 3D). As expected, we observed significant increases in Oil red O staining following 7c treatment compared to control cells, whereas 2c treatment caused an even greater amount of intracellular lipid droplets to form. Given these results, we then re‐analyzed our proteomics and phosphoproteomics datasets (Mitchell et al. 2024) to look specifically at differences in protein abundance following 7c treatment relative to 2c treatment. In this previous study, we observed significant changes to spare respiratory capacity and cell epigenetic age only with 7c. Thus, we wanted to see if we could parse out which biological pathways could be responsible for the divergent effects of the 7c and 2c cocktails. In both fibroblasts isolated from young (4‐month‐old) and old (20‐month‐old) C57BL/6J male mice, we observed a similar higher abundance of OXPHOS‐related proteins (e.g., electron transport chain, mitochondrial translation, mitochondrial organization), and a lower abundance of cell cycle‐related proteins (e.g., chromosome organization S phase, histone modification, DNA elongation), after 7c treatment relative to 2c treatment (Figure S2A). At the phosphoproteome level, we noticed key differences in the effects of 2c and 7c on chromatin organization and ion transport, particularly in old mouse fibroblasts (Figure S2B). Based on our previous observations that only 7c significantly increases cellular spare respiratory capacity (Mitchell et al. 2024), we then specifically re‐analyzed the phosphoproteomic data to assess differential phosphorylation in proteins related to the mitochondrion, lipolysis, structural muscle constituents, and the spliceosome, between 2c and 7c treatments in young and old fibroblasts (Figure S2C). We found that biological processes related to lipolysis and structural muscle constituents are more phosphorylated in 2c‐treated fibroblasts compared to 7c‐treated fibroblasts, both in young and old cells. Conversely, proteins related to the mitochondrion were significantly more phosphorylated in old 7c‐treated fibroblasts compared to old 2c‐treated fibroblasts. Therefore, we determined that the 7c and 2c cocktails differ primarily in their impact on lipid droplet formation, mitochondrial OXPHOS, and cell proliferation, which all may be interrelated.

### 3.3 Short‐Term, Low Dose 7c Treatment Does Not Strongly Impact Molecular Biomarkers of Biological Age in Male Mice

**FIGURE 4:**
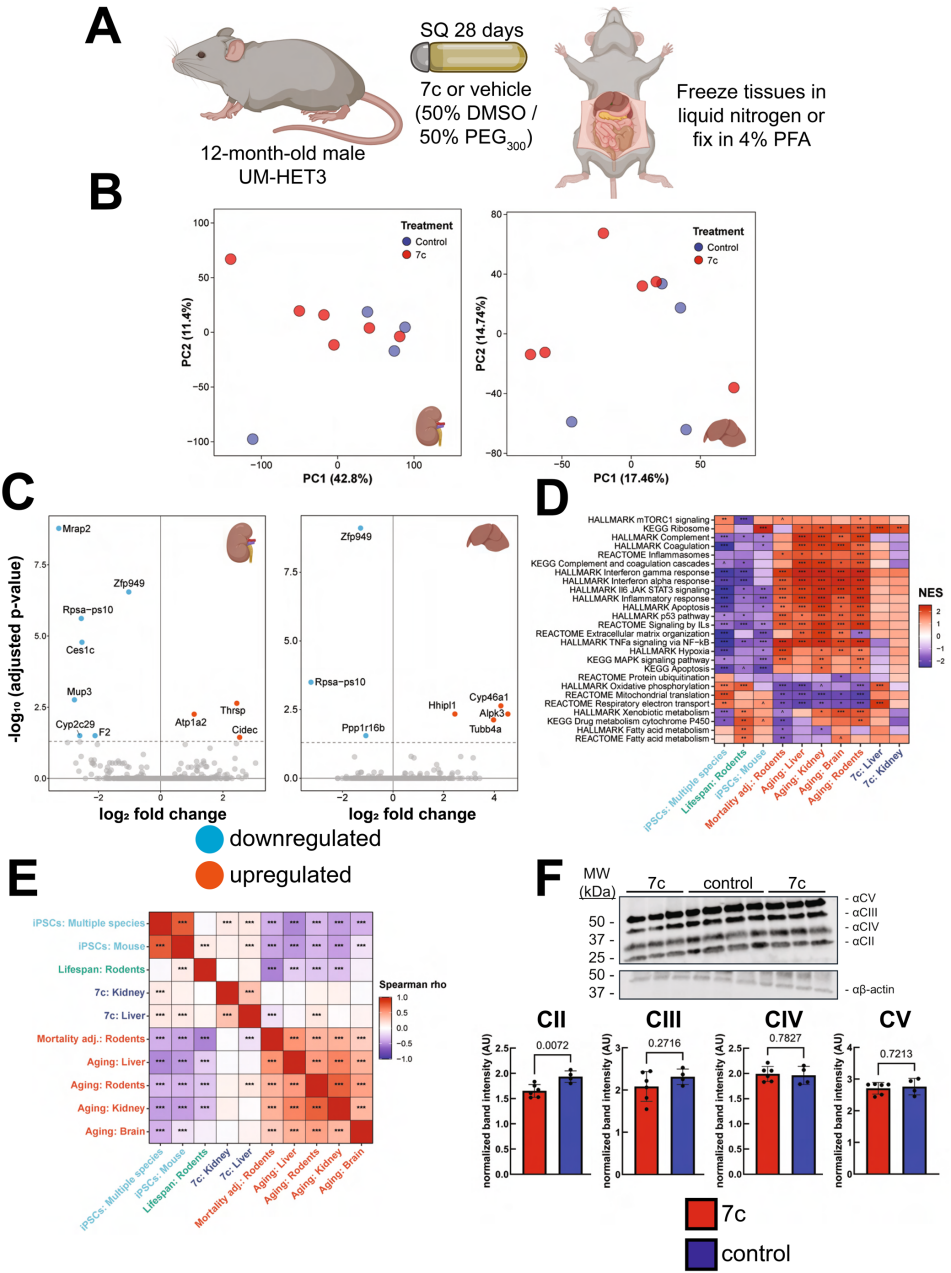
Effect of 28 days low dose 7c treatment on gene and protein expression in male UM‐HET3 mice. (A) Experimental strategy for in vivo partial chemical reprogramming. (B) Principal component analysis of bulk mRNA‐seq data. Shown here for mRNA‐seq data obtained from kidney (left) and liver (right) samples. *n* = 4–6 mice per treatment. (C) Detected differentially expressed genes following 7c treatment. Significantly (adjusted *p* < 0.05) downregulated (cyan) and upregulated (red) genes are labeled for kidney (left panel) and liver (right panel) tissues. (D) Gene set enrichment analysis (GSEA) of pathways affected by 7c treatment (dark blue). Shown here compared to signatures of aging and mortality (red), iPSCs (cyan), and expected maximum lifespan (green). Gene sets from KEGG, REACTOME, and HALLMARKS ontologies were utilized. The complete list of significantly affected functions is in Tables S4 and S5. NES: Normalized enrichment score. ^adjusted *p* < 0.1, *adjusted *p* < 0.05, **adjusted *p* < 0.01, ***adjusted *p* < 0.001. (E) Spearman correlation analysis of enriched pathways perturbed by 7c treatment (dark blue). Shown here compared to signatures of aging and mortality (red), iPSCs (cyan), and expected maximum lifespan (green). Pairwise Spearman correlations were calculated based on NES values determined for each signature via GSEA. ***adjusted *p* < 0.001. (F) Effect of 7c treatment on abundance of OXPHOS proteins. Bands for OXPHOS proteins were normalized to their respective β‐actin bands. *p*‐values were determined by two‐tailed unpaired *t*‐test. *n* = 4–6 mice per treatment.

We then sought to investigate whether 7c partial chemical reprogramming could affect molecular biomarkers of aging in a genetically heterogeneous mouse model. We chose to use the 7c cocktail because we previously showed that it robustly decreased the transcriptomic and epigenetic age of mouse fibroblasts (Mitchell et al. 2024). To address the challenge of the 7c compounds' high insolubility in water and the potential toxicity of larger amounts of DMSO administered in vivo, we developed a system for stable, long‐term delivery of the 7c cocktail over a 1‐month period using subcutaneously (SQ) implantable osmotic minipumps (Figure 4A). A solvent of 50% DMSO and 50% PEG_300_ effectively solubilized the 7c cocktail and was compatible with the pumps. Additionally, the volume of pure DMSO (which is toxic [Galvao et al. 2014; Worthley and Schott 1969]) delivered per day was less than 4 μL, and the minor surgical procedure was well‐tolerated by aged mice (Mitchell et al. 2025; Sweetwyne et al. 2017). We delivered 0.1 mg/kg/day of 6c (repsox, tranylcypromine, DZNep, forskolin, CHIR99021, TTNPB) and 1 mg/kg/day of valproate to 12‐month‐old male UM‐HET3 mice for a period of 28 days (Table S1), with control mice receiving pumps filled only with solvent. After 28 days of treatment, the mice were euthanized, and RNA extracted from their livers and kidneys were analyzed using bulk mRNA‐seq.

To evaluate the in vivo efficacy of 7c, we first utilized transcriptomic biomarkers of aging to analyze the liver and kidney bulk mRNA‐seq data. By principal component analysis (Figure 4B) of kidney (left panel) and liver (right panel) mRNA‐seq samples, we did not observe separation between 7c‐ and vehicle‐treated animals on either principal component 1 or 2. After performing a differential expression analysis (Tables S2 and S3), we observed very few differentially expressed genes in both the livers and kidneys with only a downregulation of *Zfp949* and *Rpsa‐ps10* being shared between the two tissues (Figure 4C). We then performed gene set enrichment analysis (GSEA) to determine if 7c treatment impacted the expression of gene sets associated with mammalian aging, lifespan regulation, and OSKM reprogramming (Tables S4 and S5 Figure 4D). The transcriptional changes induced by 7c in both the kidney and liver (blue) did not globally resemble the functional signatures of aging (red), iPSCs (cyan), or established lifespan‐extending interventions (green). At the level of individual pathways, the only consistently and significantly upregulated gene sets after 28 days of 7c treatment were those related to OXPHOS and ribosomal organization. We further performed a Spearman correlation analysis comparing the gene expression changes induced by 7c with reference signatures at the level of enriched functions (Figure 4E). Transcriptional changes induced by 7c in the liver exhibited modest positive correlation with iPSCs and rodent aging signatures, and a negative correlation with signatures of rodent mortality. Furthermore, we were unable to detect an impact of 7c treatment on kidney or liver transcriptomic age using either rodent chronological or mortality transcriptomic clocks (Figure S3). Although OXPHOS genes were positively enriched at the transcriptomic level in the liver, we did not observe a corresponding increase in OXPHOS protein abundance after 28 days with 7c treatment (Figure 4F). Thus, we concluded that at this first dosage tested, 7c treatment is safely administered but does not lead to robust changes in mitochondrial OXPHOS or transcriptomic biomarkers of biological age.

### 3.4 High Dose 7c Treatment Causes Excessive Lipid Accumulation and Toxicity in Male Mice

**FIGURE 5:**
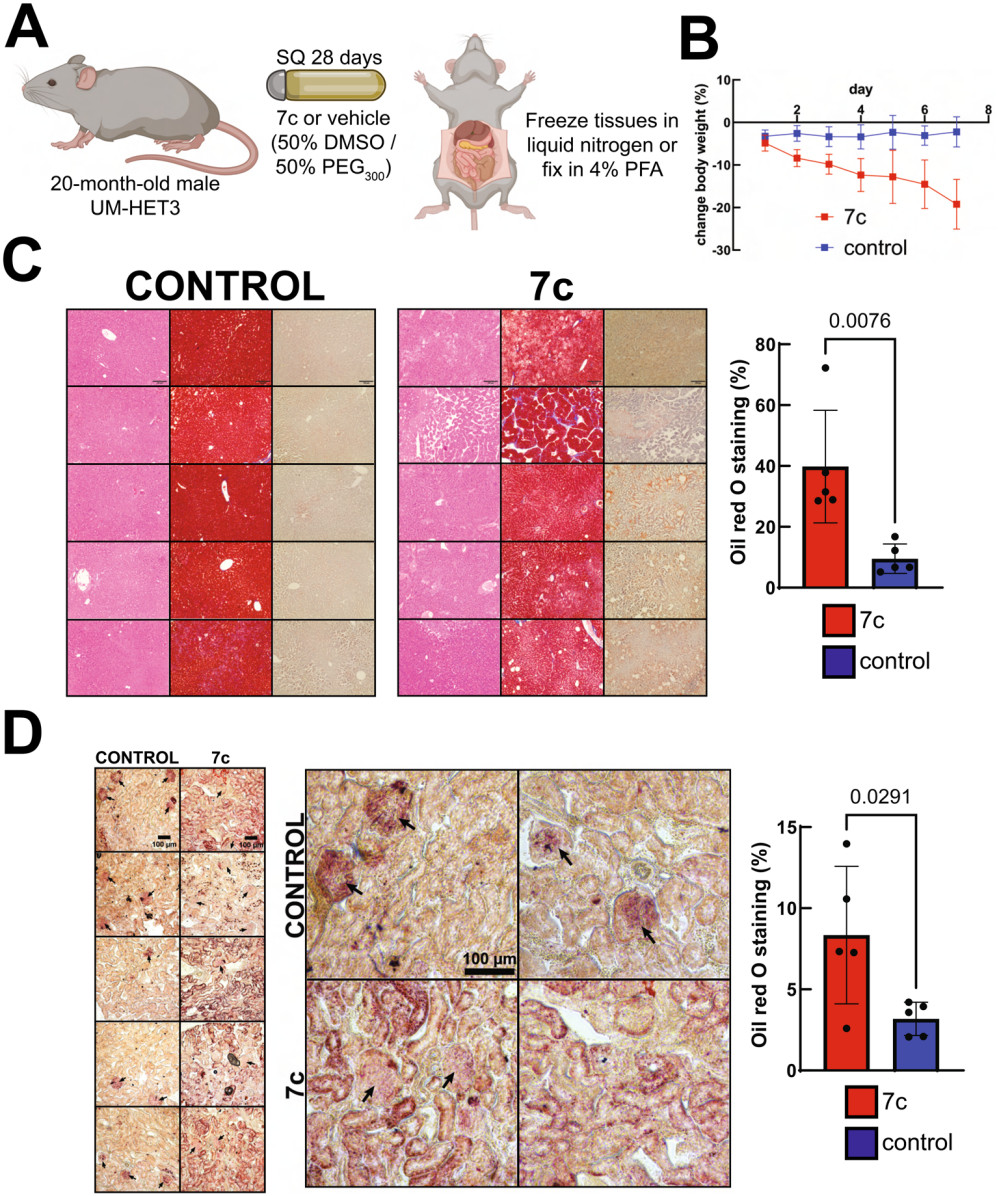
Effect of high dose 7c treatment in male UM‐HET3 mice. (A) Experimental strategy for in vivo partial chemical reprogramming. (B) Effect of high dose 7c treatment on body weight. Data points represent sample means ± SD. *n* = 6 mice per treatment. (C) Effect of high dose 7c treatment on liver histology. Liver sections were stained with hematoxylin and eosin (left panels), Masson's trichrome (center panels), and Oil red O (right panels). Each row represents images obtained from stained tissues of a single animal. Scale bars: 100 μm. *n* = 5 mice per treatment. Error bars represent sample means ± SD. *p*‐value was determined by two‐tailed unpaired *t*‐test. (D) Effect of high dose 7c treatment on kidney histology. Kidney sections were stained with Oil red O. Each panel represents an image obtained from stained tissue of a single animal. Scale bars: 100 μm. Arrows indicate glomeruli. *n* = 5 mice per treatment. *p*‐value was determined by two‐tailed unpaired *t*‐test.

Since we didn't observe any evidence of lowered transcriptomic age or upregulation of mitochondrial OXPHOS with this first treatment regiment, we decided to increase the dosage to 0.5 mg/kg/day for 6c and 50 mg/kg/day for valproate (Figure 5A). However, this dose resulted in a rapid decrease in body weight and body condition scores in ~5–6 days that required euthanasia per institutional guidelines. A follow‐up trial using an intermediate dosage of 0.25 mg/kg/day for 6c and 10 mg/kg/day for valproate similarly resulted in a significant decrease in body weight after 7 days (Figure 5B). Gross necropsy did not reveal any obvious features (e.g., tumors, hemorrhages, liver failure) that may have contributed to the drug‐induced toxicity. Therefore, we then sectioned the fixed liver and kidney tissues and stained both with hematoxylin and eosin, Masson's trichrome, and Oil red O. In the livers (Figure 5C) of animals treated with 7c, we did not observe increased collagen staining indicative of fibrosis, or any apoptotic bodies being formed (Parras et al. 2023). However, we did notice a strong increase in Oil red O staining that was further validated by quantification of the stained area. Similarly, we did not observe an increase in fibrosis in the kidneys of the 7c‐treated animals (Figure S4) but also saw a strong increase in overall Oil red O staining (Figure 5D). In the animals treated with vehicle, we observed age‐related glomerular changes and positive lipid staining (black arrows). However, for the animals treated with 7c, we noticed a significant decrease of Oil red O staining in the glomeruli. Therefore, we concluded that 7c treatment can cause the accumulation of triglycerides in multiple organs and alter kidney microscopic anatomy.

**FIGURE 6:**
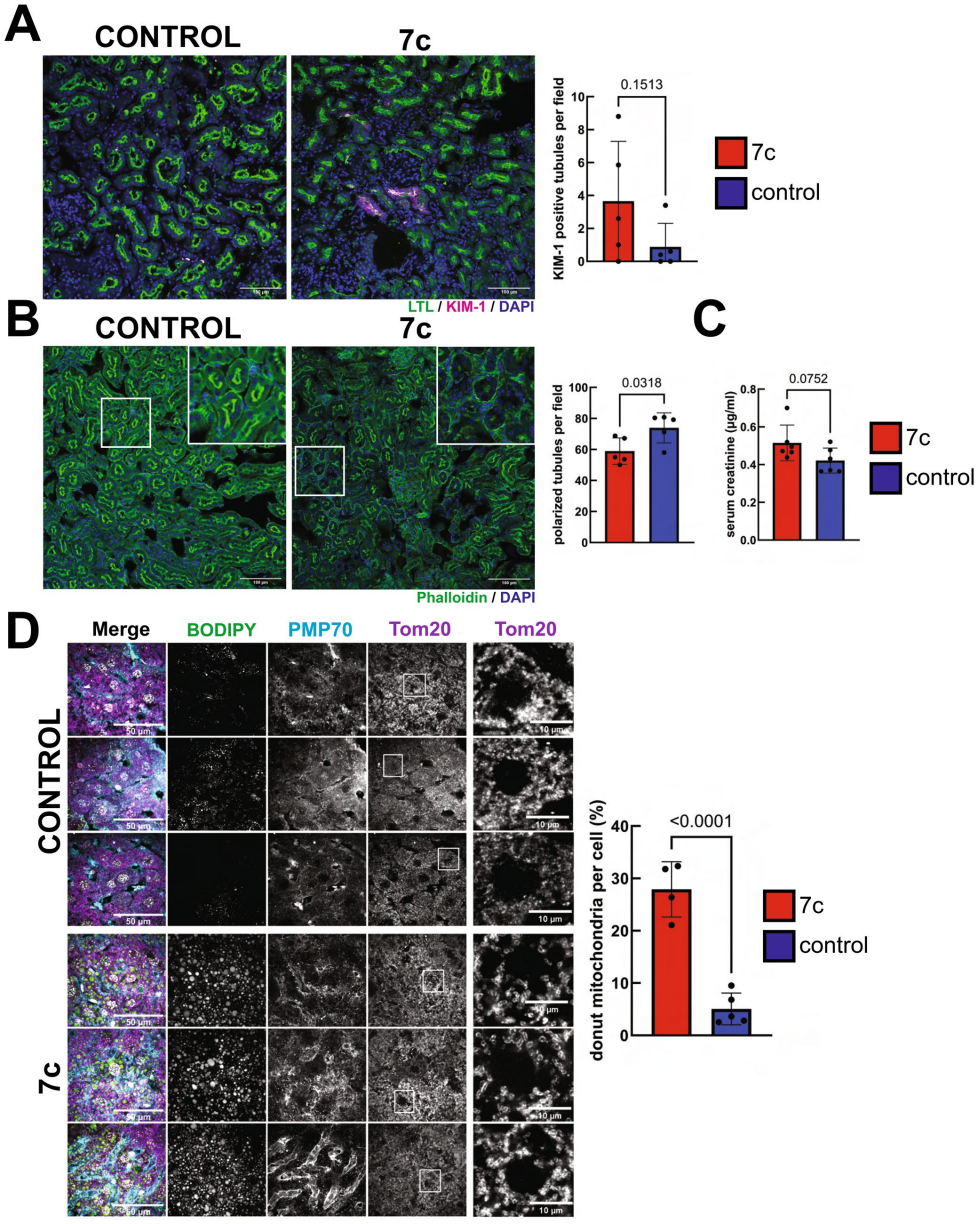
Effect of high dose 7c treatment on markers of acute kidney and liver injury. (A) Effect on renal tubular injury. Representative images of KIM‐1‐ and LTL‐stained kidney sections from control and 7c‐treated mice. Five to seven images were acquired per mouse. *p*‐values were determined by two‐tailed unpaired *t*‐test. *n* = 5 mice per treatment. Error bars represent sample means ± SD. Scale bars: 100 μm. (B) Effect on renal tubular polarization. Representative images of phalloidin‐stained kidney sections from control and 7c‐treated mice. Five to nine images were acquired per mouse. *p*‐values were determined by two‐tailed unpaired *t*‐test. *n* = 5 mice per treatment. Error bars represent sample means ± SD. Scale bars: 100 μm. (C) Effect on serum creatinine levels. *p*‐value was determined by two‐tailed unpaired *t*‐test. *n* = 6 mice per treatment. Error bars represent sample means ± SD. (D) Effect on mitochondrial morphology in liver. Representative images of PMP70‐, BODIPY‐, and Tom20‐stained liver sections from control and 7c‐treated mice. Three *z*‐stacks were acquired per mouse. *p*‐values were determined by two‐tailed unpaired *t*‐test. *n* = 4–5 mice per treatment. Error bars represent sample means (percent of total mitochondria per cell exhibiting donut‐like morphologies) ± SD. Merged scale bars: 50 μm. Tom20 inset scale bars: 10 μm.

Due to the changes we observed in glomeruli lipid staining following 7c treatment, we then evaluated 7c treatment for its impact on markers of acute renal injury. We first stained kidney sections for LTL, which is a general marker for proximal tubule brush border integrity (Hennigar et al. 1985), and KIM‐1 (Figure 6A), which is upregulated in the proximal tubule after injury/ischemia (Ichimura et al. 1998). In kidneys from control animals, very few proximal tubules were positive for KIM‐1 expression. Although two 7c‐treated animals showed pronounced increases in KIM‐1‐stained proximal tubules, the effect across the entire treatment group was not significant. However, when we stained with phalloidin, we noticed a significant loss in polarized tubules with apical F‐actin in the 7c‐treated animals (Figure 6B). It has been previously reported that the cytoskeleton of proximal tubules is disrupted after ischemia (Kellerman and Bogusky 1992). Finally, to assess overall kidney function, we measured serum creatinine levels using mass spectrometry (Figure 6C). Creatinine levels were generally low across both treatment groups (Takahashi et al. 2007); however, serum creatinine levels in the 7c‐treated animals were slightly elevated with marginal statistical significance. Therefore, we concluded that high dose 7c treatment can cause acute injury reminiscent of ischemia.

Finally, to gain additional insights into the potential source of the increased lipid accumulation following 7c treatment, we stained liver sections for peroxisomes (PMP70), lipid droplets (BODIPY 493/503), and mitochondria (Tom20) (Figure 6D) (Garcia et al. 2023; Imanaka et al. 1999). The staining with BODIPY 493/503 revealed a drastic increase in lipid droplet size for the 7c‐treated animals, consistent with the results obtained from staining tissues with Oil red O. While no discernible differences in peroxisome morphology or abundance were consistent across biological replicates, 7c‐treated animals demonstrated an increase in liver mitochondria with donut‐like morphologies. These atypical morphologies have been previously reported in the liver and are suggested to form in response to metabolic stress and/or opening of the permeability transition pore (Jenkins et al. 2024; Lionetti et al. 2014; Liu and Hajnóczky 2011). Taken together, our results suggest that metabolic stress induced by 7c treatment induces lipid droplet accumulation in liver and kidneys and contributes to acute kidney injury in vivo.

## 4 Discussion

We assessed the effects of 7c partial chemical reprogramming on mitochondrial morphology and dynamics in mouse fibroblasts and measured the impact of 7c treatment on organismal biological age and health using genetically diverse UM‐HET3 male mice. Consistent with previous reports, we have determined that 7c partial chemical reprogramming causes significant changes to mitochondrial interaction networks, movement dynamics, and morphology. In tandem, we observed that treatment with both 7c and 2c causes a significant increase in lipid droplet formation in vitro, with 2c treatment having an even more pronounced effect compared to 7c treatment in fibroblasts. This result was further supported by our previous phosphoproteomics data, which demonstrated that, relative to 7c treatment, 2c treatment significantly dephosphorylates proteins involved in lipolysis. When we treated male UM‐HET3 mice with a low dose of the 7c cocktail for 28 days, we did not observe significant effects on kidney or liver transcriptomic age, overall gene expression, or on the abundance of mitochondrial OXPHOS proteins. Higher doses of 7c, in contrast, led to a rapid loss of body weight and lowered body condition scores that required euthanasia. Therefore, partial chemical reprogramming currently seems unable to induce more youthful gene expression profiles in mammalian tissues without causing toxicity.

While Masson's trichrome and hematoxylin and eosin staining did not reveal the presence of fibrosis or apoptotic bodies, we did observe significant lipid accumulation in the livers (similar to early‐stage steatosis (El‐Zayadi 2008)) and kidneys of 7c‐treated animals, which were consistent with our observations in vitro. We also noticed a slight elevation in biomarkers of acute kidney injury, including cytoskeletal changes in proximal tubules and some 7c‐treated animals with increased proximal tubule KIM‐1 expression. Thus, our current research suggests that this rapid and severe accumulation of intracellular lipids during partial chemical reprogramming may contribute to the signs of toxicity we observed in our mouse models. Moreover, we have uncovered evidence that partial chemical reprogramming with 7c contributes to mitochondrial stress both in vitro and in vivo, as noted by the decreases in cellular ATP and NAD^+^/NADH, and the increase in donut‐shaped mitochondria, respectively. Previously, we reported that 7c treatment gradually increases the number of apoptotic cells, as well as decreases the abundance of several TCA cycle metabolites (e.g., succinic acid, citric acid, fumaric acid, malic acid) (Mitchell et al. 2024). Therefore, our results suggest that changes in mitochondrial function induced by 7c treatment may promote the accumulation of lipid droplets. It has been previously reported that mitochondrial stress upregulates fatty acid biosynthesis; however, in this context, cells switch metabolism from oxidative phosphorylation to glycolysis to attempt to mitigate the associated stress (Lee et al. 2013). Thus, it is unusual that partial chemical reprogramming causes an increase in lipid droplet accumulation, which is meant to be protective against metabolic stress (Jarc and Petan 2019), despite inducing a strong oxidative phosphorylation phenotype (Mitchell et al. 2024).

It is also important to note the strong differences between reprogramming and chemical reprogramming. On one hand, gene expression signatures of OSKM reprogramming reveal a suppression of mesenchymal genes, increased proliferation, and a metabolic switch to glycolysis during the early phases of reprogramming (Smith et al. 2016). Moreover, there is decreased and increased expression of genes related to inflammation and DNA repair, respectively (Kriukov et al. 2022). Despite these transcriptional changes, the epigenome and histone trimethylation levels of somatic cells are largely maintained until later in the reprogramming process when the expression of epithelial genes is activated (Smith et al. 2016). On the other hand, chemical reprogramming is marked by global demethylation along with an early transition to a plastic extraembryonic endoderm (XEN)‐like cells during stage I (Zhao et al. 2018). This occurs in tandem with upregulated OXPHOS, increased reactive oxygen species production, and reduced cell proliferation (Mitchell et al. 2024; Schoenfeldt et al. 2022). Notably, the expression of most pluripotency markers is not activated until stage II, in which *Oct4* activation occurs simultaneously with a gradual downregulation of XEN genes (Mitchell et al. 2024; Zhao et al. 2018). Therefore, these data suggest that partial reprogramming and partial chemical reprogramming rejuvenate cells through entirely disparate mechanisms and, as a result, may present distinct advantages and challenges for their applications in vivo.

Significant evidence exists that supports the interaction of mitochondria with lipid droplets. These interactions may be crucial for regulating fatty acid metabolism and overall energy homeostasis under both basal and stress conditions (Cui and Liu 2020; Fan and Tan 2024; Talari et al. 2023). Thus, in the case of partial chemical reprogramming with 7c, it may be that the strong increase in lipid droplet formation is necessary to mediate the increase in mitochondrial bioenergetic capacity, and vice versa. With 2c, lipid accumulation in contrast is higher, which may make this cocktail even more toxic in vivo, yet it does not lead to robust changes in mitochondrial OXPHOS function. While current data cannot causally relate the increase in mitochondrial OXPHOS following 7c partial chemical reprogramming to the reduction in cellular transcriptomic and epigenetic age, we do note that the upregulation of mitochondrial/OXPHOS genes contributes significantly to the observed decrease in transcriptomic age induced by this cocktail in cells (Figure S5). Given that the lipid droplets being formed during partial chemical reprogramming may contribute to its toxicity, one might assume that modification and/or removal of the compound(s) primarily responsible for this effect could lead to a safer cocktail that can also lower biological age in vivo. However, the possibility cannot be ignored that the lowered cellular biological age in response to 7c treatment may be dependent on this increase in lipid droplet formation. Therefore, further experimentation is required to determine whether or not the rejuvenating and toxic effects of partial chemical reprogramming can be separated.

## 5 Limitations and Future Directions

The in vivo studies described herein were limited in the number of biological replicates and to only one sex. Therefore, it is unknown if 7c treatment would produce similar effects in female mice. For future studies, it would be important to determine which compound(s) are primarily responsible for the observed toxic effects, and to see if a cocktail without these compound(s) could still have pronounced effects on cell biological age. Finally, we have not performed any in vitro testing of the 7c cocktail in epithelial cells. Therefore, we acknowledge that the observed in vivo toxicity of the treatment could stem from injury to epithelial cells.

## Author Contributions

W.M., V.N.G. conceptualization; W.M., C.G.M., A.T., Y.U., L.J.E.G., T.I., E.L.N., J.V.B., V.N.G. methodology; W.M., C.G.M., Y.U. data curation; W.M., C.G.M., A.T., Y.U., L.J.E.G., T.I. formal analysis; W.M., C.G.M., A.T., Y.U., L.J.E.G., T.I., E.L.N., A.M. validation; W.M., C.G.M., A.T., Y.U., L.J.E.G. visualization; W.M., writing – original draft; W.M., C.G.d.M., A.T., Y.U., L.J.E.G., E.L.N., T.I., A.M., J.V.B., V.N.G. writing – review and editing; J.V.B., V.N.G. supervision; J.V.B., V.N.G. project administration; V.N.G. funding acquisition.

## Funding

W.M. was supported by a T32 fellowship (grant number EB016652) from NIH‐NIBIB. This work was supported by NIH‐NIA grants to V.N.G., NIH‐NIDDK grants R01DK39773 and R01DK072381 to J.V.B., and by funds from the James Fickel Foundation.

## Conflicts of Interest

The authors declare no conflicts of interest.

## Supporting information


**Data S1:** acel70390‐sup‐0001‐Supinfo.pdf.

## Data Availability

mRNA‐seq data is accessible via GEO series GSE300625. All other supporting data is available as supplemental data files and/or upon request from the corresponding author.
