# Supplementary material for: In Vivo Chemical Reprogramming Is Associated With a Toxic Accumulation of Lipid Droplets Hindering Rejuvenation
Source: Aging Cell. 2026 Jan 27;25(2):e70390. doi: 10.1111/acel.70390 (PMC12835892; doi:10.1111/acel.70390)
Supplement: Supplementary file 1 — Data S1: acel70390‐sup‐0001‐Supinfo.pdf. [file ACEL-25-e70390-s001.pdf]

Supporting Material for

In vivo chemical reprogramming is associated with a toxic accumulation of lipid droplets hindering rejuvenation

Wayne Mitchell[1], Cecília G. de Magalhães[1], Alexander Tyshkovskiy[1], Yushi Uchida[2], Ludger J.E. Goeminne[1], Takaharu Ichimura[2], Emery L. Ng[3], Alibek Moldakozhayev[1], Joseph V. Bonventre[2], and Vadim N. Gladyshev[1*]

[1]Division of Genetics, Department of Medicine, Brigham and Women's Hospital, Harvard Medical School, Boston, MA 02115 United States
[2]Division of Renal Medicine, Brigham and Women's Hospital, Boston, MA 02115 United States
[3]Fralin Life Sciences Institute, Virginia Tech, Blacksburg, VA 24061 United States

Table of contents:

Table S1 – Drug supplier and dosage information

Table S2 – Liver differentially expressed genes

Table S3 – Kidney differentially expressed genes

Table S4 – Significant liver GSEA terms

Table S5 – Significant kidney GSEA terms

Supplemental Figure S1 – Effect of uncoupler treatment on mitochondrial morphology and networks

Supplemental Figure S2 – Comparison of 7c vs. 2c treatment on protein and phosphoprotein abundance

Supplemental Figure S3 – Effect of *in vivo* partial chemical reprogramming on liver and kidney tissue transcriptomic age

Supplemental Figure S4 – Additional kidney histology images

Supplemental Figure S5 – Modular transcriptomic age of fibroblasts treated with 2c and 7c

Table S1: Drug supplier and dosage information

Compound	Supplier	Catalog #	Cell Culture Concentration (μM)	Trial 1 Dose (mg/kg/day)	Trial 2 Dose (mg/kg/day)	Trial 3 Dose (mg/kg/day)
Valproate	STEMCELL Technologies	72292	500	1	50	10
Repsox	Sigma-Aldrich	R0158	5	0.1	0.5	0.25
Tranylcypromine	Sigma-Aldrich	P22370	5	0.1	0.5	0.25
Forskolin	Tocris	1099	10	0.1	0.5	0.25
CHIR99021	Cayman Chemical	13122	10	0.1	0.5	0.25
DZNep	APExBIO	A1905	0.5	0.1	0.5	0.25
TTNPB	Cayman Chemical	16144	1	0.1	0.5	0.25

Table S1

Table S2: Liver differentially expressed genes

Entrez ID	Gene Symbol	log2FC	P-value	adjusted P-value
71640	Zfp949	-1.282	5.94E-14	7.82E-10
100040633	Rpsa-ps10	-3.240	4.83E-08	3.18E-04
13116	Cyp46a1	4.259	5.25E-07	2.30E-03
116904	Alpk3	4.536	1.53E-06	4.57E-03
214305	Hhipl1	2.443	1.74E-06	4.57E-03
22153	Tubb4a	3.971	3.40E-06	7.45E-03
228852	Ppp1r16b	-1.073	1.49E-05	2.80E-02

Table S2

Table S3: Kidney differentially expressed genes

Entrez ID	Gene Symbol	log2FC	P-value	adjusted P-value
244958	Mrap2	-3.274	1.11E-13	1.67E-09
71640	Zfp949	-1.025	3.78E-11	2.85E-07
100040633	Rpsa-ps10	-2.551	4.84E-10	2.43E-06
13884	Ces1c	-2.529	4.40E-09	1.66E-05
17842	Mup3	-2.771	5.76E-07	1.74E-03
21835	Thrsp	2.448	9.15E-07	2.30E-03
98660	Atp1a2	1.077	2.58E-06	5.56E-03
13095	Cyp2c29	-2.600	1.78E-05	3.16E-02
14061	F2	-2.108	1.88E-05	3.16E-02
14311	Cidec	2.538	2.39E-05	3.61E-02

Table S3

Table S4: Significant liver GSEA terms

Term	NES	P-value	adjusted P-value
REACTOME SRP DEPENDENT COTRANSLATIONAL PROTEIN TARGETING TO MEMBRANE	2.193	2.84E-08	2.63E-05
REACTOME RESPIRATORY ELECTRON TRANSPORT	2.187	4.68E-08	2.63E-05
REACTOME RESPIRATORY ELECTRON TRANSPORT ATP SYNTHESIS BY CHEMIOSMOTIC COUPLING AND HEAT PRODUCTION BY UNCOUPLING PROTEINS	2.173	7.43E-08	2.78E-05
REACTOME CELLULAR RESPONSE TO STARVATION	2.019	1.70E-07	4.76E-05
REACTOME SELENOAMINO ACID METABOLISM	2.141	4.04E-07	9.07E-05
REACTOME RESPONSE OF EIF2AK4 GCN2 TO AMINO ACID DEFICIENCY	2.166	4.90E-07	9.16E-05
KEGG RIBOSOME	2.183	6.70E-07	9.61E-05
REACTOME THE CITRIC ACID TCA CYCLE AND RESPIRATORY ELECTRON TRANSPORT	1.975	6.85E-07	9.61E-05
REACTOME METABOLISM OF AMINO ACIDS AND DERIVATIVES	1.763	7.89E-07	9.85E-05
REACTOME EUKARYOTIC TRANSLATION ELONGATION	2.130	2.41E-06	2.47E-04
REACTOME TRANSLATION	1.778	2.47E-06	2.47E-04
HALLMARK OXIDATIVE PHOSPHORYLATION	1.894	2.64E-06	2.47E-04
REACTOME REGULATION OF EXPRESSION OF SLITS AND ROBOS	1.846	1.10E-05	9.51E-04
KEGG OXIDATIVE PHOSPHORYLATION	2.018	2.31E-05	1.85E-03
REACTOME SELECTIVE AUTOPHAGY	1.941	6.00E-05	4.49E-03
REACTOME COMPLEX I BIOGENESIS	1.986	8.28E-05	5.81E-03
REACTOME INFLUENZA INFECTION	1.764	9.28E-05	6.13E-03
REACTOME AGGREPHAGY	2.064	1.35E-04	8.39E-03
REACTOME MHC CLASS II ANTIGEN PRESENTATION	1.809	1.42E-04	8.39E-03
REACTOME HSP90 CHAPERONE CYCLE FOR STEROID HORMONE RECEPTORS SHR IN THE PRESENCE OF LIGAND	1.977	1.88E-04	1.05E-02
HALLMARK ALLOGRAFT REJECTION	1.701	2.50E-04	1.22E-02
KEGG PARKINSONS DISEASE	1.890	2.34E-04	1.22E-02
KEGG PRIMARY IMMUNODEFICIENCY	1.983	2.46E-04	1.22E-02
REACTOME NONSENSE MEDIATED DECAY NMD	1.821	2.69E-04	1.26E-02
REACTOME GAP JUNCTION ASSEMBLY	1.925	3.37E-04	1.51E-02
REACTOME SIGNALING BY ROBO RECEPTORS	1.660	3.49E-04	1.51E-02
KEGG ANTIGEN PROCESSING AND PRESENTATION	1.892	3.75E-04	1.56E-02
REACTOME ROS AND RNS PRODUCTION IN PHAGOCYTES	1.952	4.01E-04	1.61E-02
REACTOME EUKARYOTIC TRANSLATION INITIATION	1.790	4.17E-04	1.62E-02
REACTOME AUTOPHAGY	1.641	6.42E-04	2.40E-02
REACTOME CELLULAR RESPONSE TO CHEMICAL STRESS	1.650	6.62E-04	2.40E-02
HALLMARK ADIPOGENESIS	1.614	7.84E-04	2.70E-02
REACTOME POST CHAPERONIN TUBULIN FOLDING PATHWAY	1.870	7.93E-04	2.70E-02
REACTOME THE ROLE OF GTSE1 IN G2 M PROGRESSION AFTER G2 CHECKPOINT	1.762	9.42E-04	3.02E-02
KEGG SYSTEMIC LUPUS ERYTHEMATOSUS	1.822	9.22E-04	3.02E-02
KEGG GLYCOLYSIS GLUCONEOGENESIS	1.833	1.02E-03	3.19E-02
REACTOME CYTOPROTECTION BY HMOX1	1.630	1.16E-03	3.43E-02
KEGG HUNTINGTONS DISEASE	1.627	1.16E-03	3.43E-02

Table S4

Table S5: Significant kidney GSEA terms

Term	NES	P-value	adjusted P-value
KEGG RIBOSOME	1.979	1.50E-05	8.36E-03
HALLMARK MYOGENESIS	1.904	3.38E-06	3.91E-03
REACTOME EUKARYOTIC TRANSLATION ELONGATION	1.992	2.17E-05	8.36E-03
KEGG LINOLEIC ACID METABOLISM	-2.020	4.69E-05	1.35E-02

Table S5

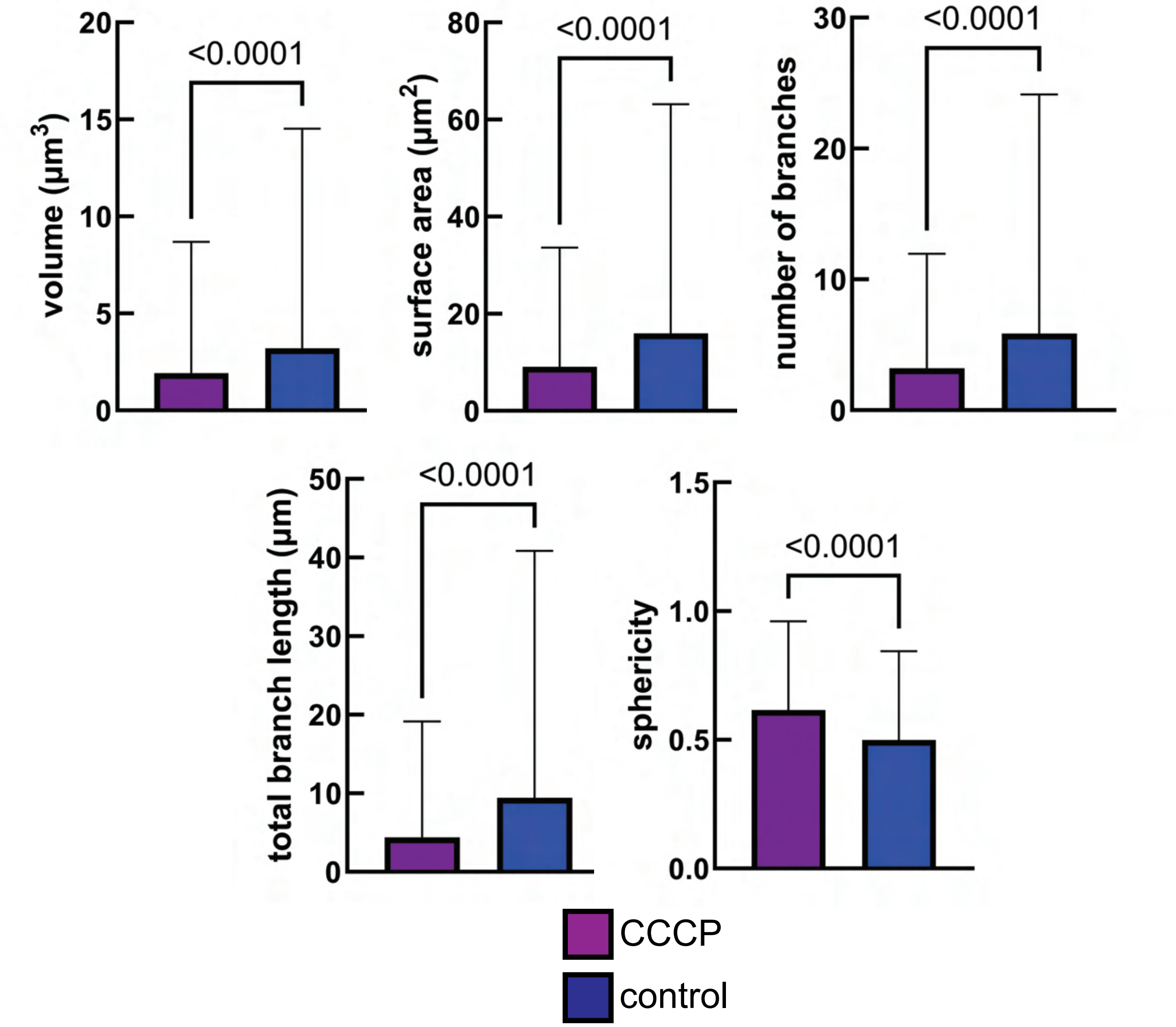

Supplemental Figure S1

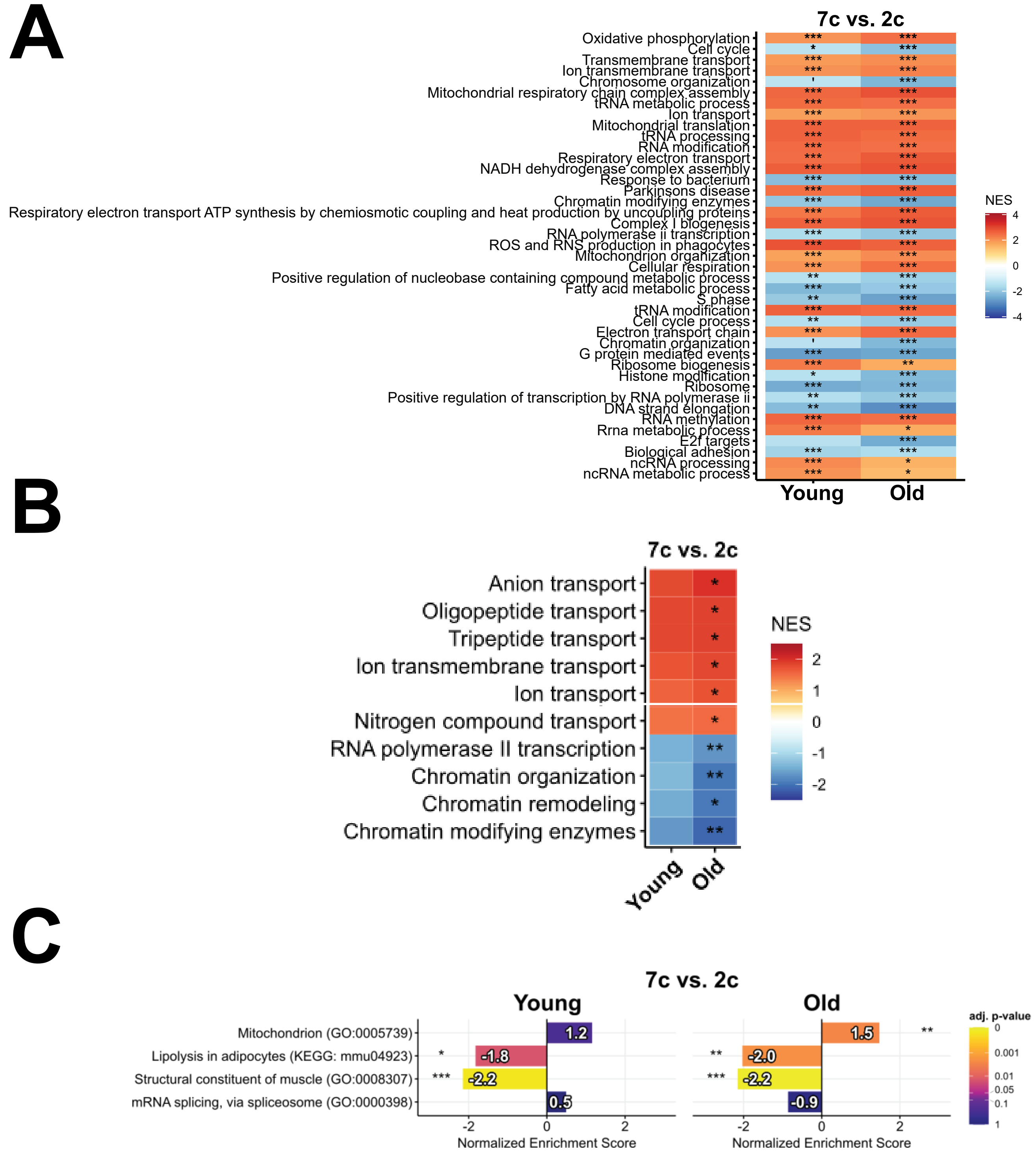

Supplemental Figure S2

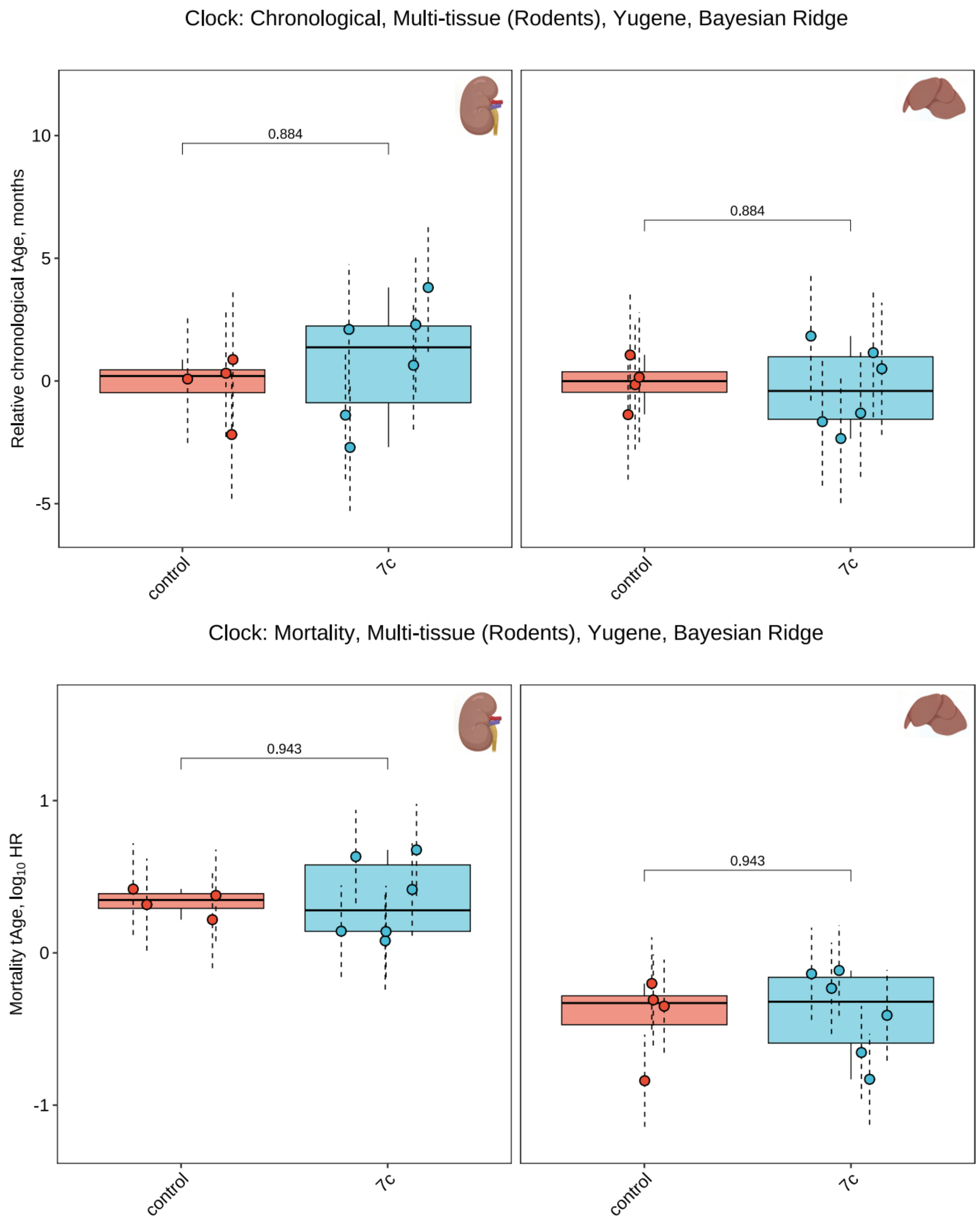

Supplemental Figure S3

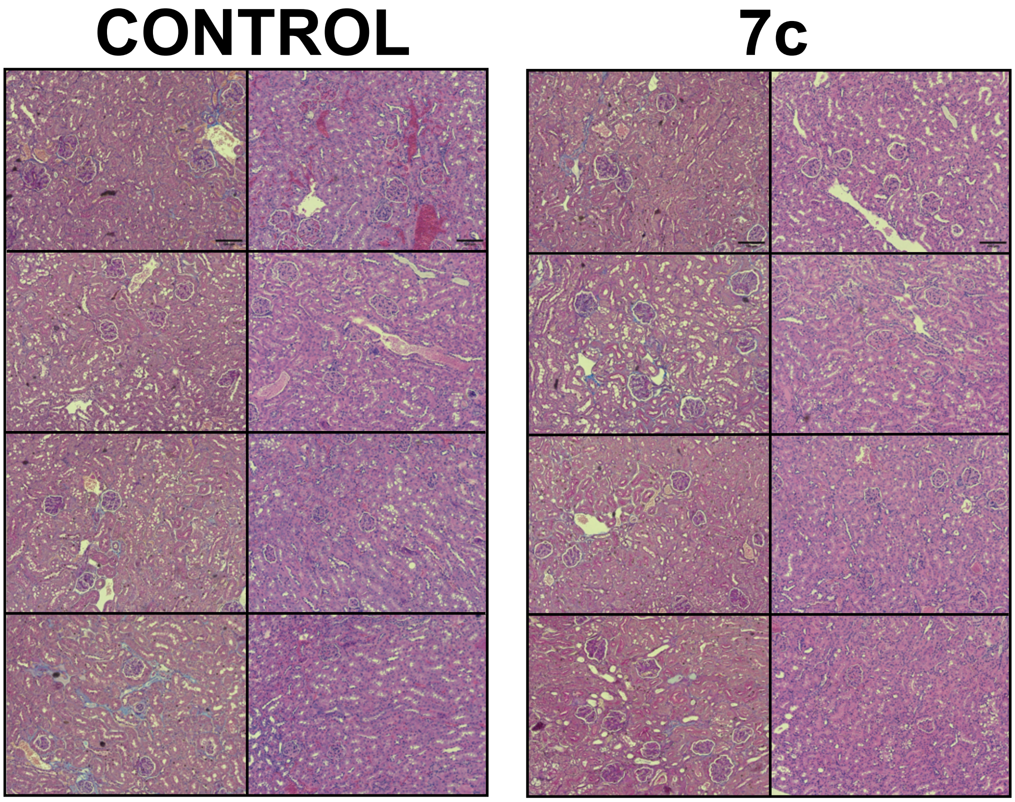

Supplemental Figure S4

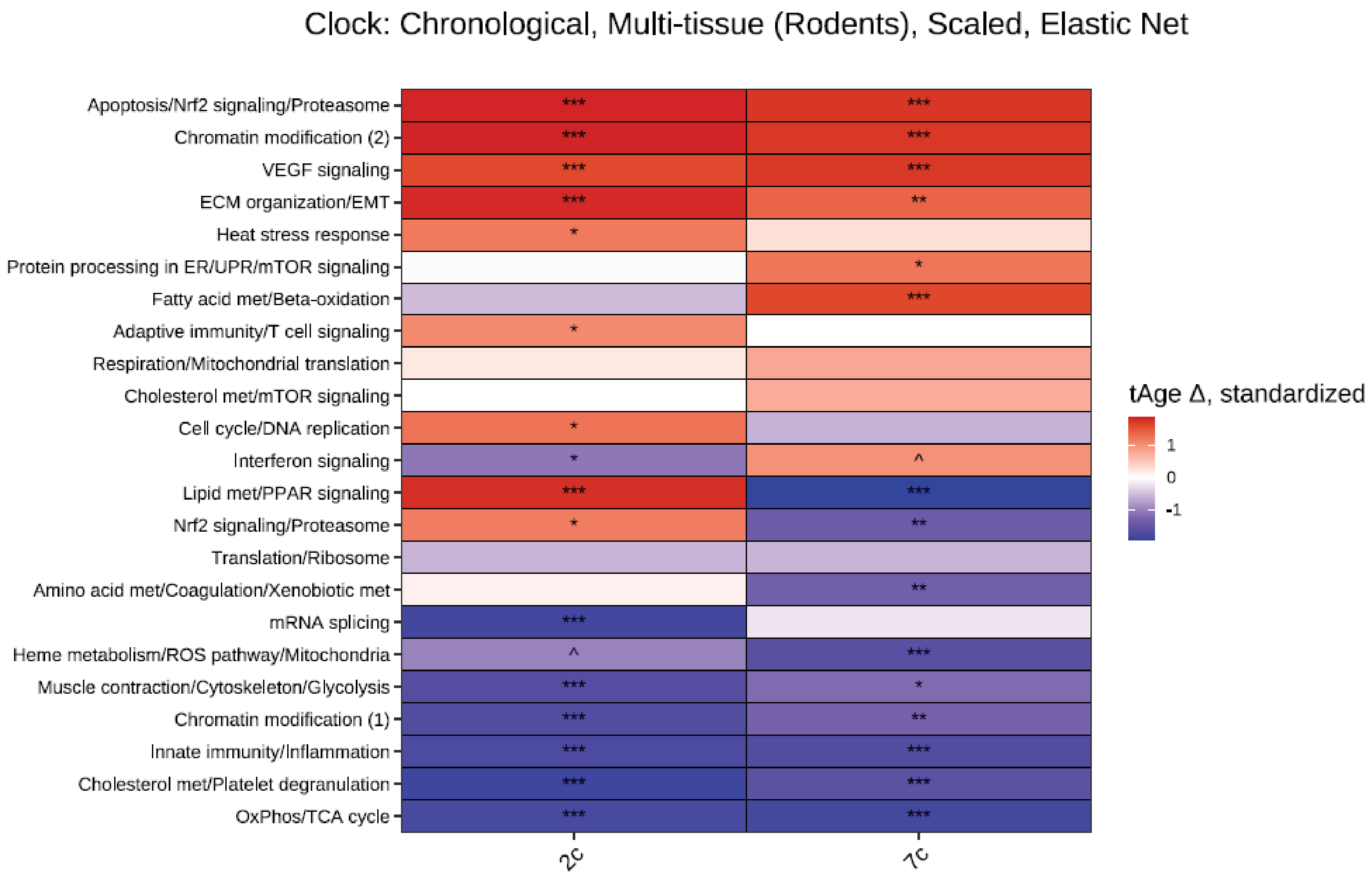

Supplemental Figure S5
